# Effects of Short- and Medium-Chain Fatty Acids on Production, Meat Quality, and Microbial Attributes—A Review

**DOI:** 10.3390/molecules28134956

**Published:** 2023-06-23

**Authors:** Rubina Tünde Szabó, Mária Kovács-Weber, Ágnes Zimborán, Levente Kovács, Márta Erdélyi

**Affiliations:** 1 Institute of Animal Husbandry, Gödöllő Campus, Hungarian University of Agriculture and Life Sciences, H-2100 Gödöllő, Hungary; szabo.rubina.tunde@uni-mate.hu (R.T.S.); kovacs-weber.maria@uni-mate.hu (M.K.-W.); zimboranagnes@gmail.com (Á.Z.); 2 Department of Feed Toxicology, Institute of Physiology and Nutrition, Gödöllő Campus, Hungarian University of Agriculture and Life Sciences, H-2100 Gödöllő, Hungary; ballane.erdelyi.marta@uni-mate.hu

**Keywords:** SCFA, MCFA, broilers, poultry, gut health, antimicrobial effect

## Abstract

The non-therapeutic use of antimicrobials in poultry production contributes to the spread of drug-resistant pathogens in both birds and humans. Antibiotics are known to enhance feed efficiency and promote the growth and weight gain of poultry. New regulatory requirements and consumer preferences have led to a reduced use of antibiotics in poultry production and to the discovery of natural alternatives to antibiotic growth promoters. This interest is not only focused on the direct removal or inhibition of causative microorganisms but also on the prevention of diseases caused by enteric pathogens using a range of feed additives. A group of promising feed additives is composed of short- and medium-chain fatty acids (SCFAs and MCFAs) and their derivatives. MCFAs possess antibacterial, anticoccidial, and antiviral effects. In addition, it has been proven that these acids act in synergy if they are used together with organic acids, essential oils, or probiotics. These fatty acids also benefit intestinal health integrity and homeostasis in broilers. Other effects have been documented as well, such as an increase in intestinal angiogenesis and the gene expression of tight junctions. The aim of this review is to provide an overview of SCFAs and MCFAs as alternatives to antibiotic growth promoters and to summarize the current findings in the literature to show their possible benefits on production, meat quality, and gut health in poultry.

## 1. Introduction

Organic acids as feed additives are common subjects of research in poultry nutrition due to food safety aspects, particularly with respect to lowering the incidence of foodborne pathogens in poultry. Research most often pays attention to particular lipid groups (saturated, unsaturated, polyunsaturated fats, or fats grouped by the length of their fatty acid chains into short-, medium-, or long-chain fatty acids). It has been proven that short- and medium-chain fatty acids (SCFAs and MCFAs, respectively) have positive effects on health, production, feed digestibility, and lower body and muscle fats in broilers [1].

The non-therapeutic use of antibiotics increases the rate of weight gain and/or the efficiency of feed utilization in food animals [2]. Since the 1980s, antibiotic growth promoters (AGPs) have been utilized widely in poultry diets to improve performance and feed conversion [3]. They have also been utilized to protect animals from the adverse effects of enteric microorganisms [4], as well as to modulate inflammation [5].

The trend toward banning the use of AGPs in poultry nutrition has been developing since the early 2000s because consumers are becoming increasingly concerned about the nutritional and health aspects of their food, as these compounds contribute to the spread of antibiotic resistance in microbes, as well as to the presence of antibiotic residues in poultry products [6,7,8]. The removal of antibiotics from compound feeds, after they were banned by the European Union in 2006, has put great pressure on poultry producers to find alternatives to antibiotics to improve gut health and growth performance [9,10,11,12]. As alternatives to AGPs, MCFAs are widely used as feed additives in broiler diets as natural sources to inhibit bacterial growth and enteric pathogen colonization [13,14]. There are several hypotheses in the literature on the antimicrobial activity of these fatty acids. One is based on the basic principle that undissociated organic acids (non-ionized and more lipophilic) can penetrate the bacterial cell wall, triggering an ionization of fatty acids, which results in the disruption of the normal physiology of certain types of bacteria [15,16,17], including increases in the fluidity, solubility, permeability, and instability of the bacterial membrane [18,19]. There is another theory about modifying gene expression in pathogens, as MCFAs are proven to be involved in the downregulation of certain genes (e.g., hil A and hil D) that are responsible for virulence [20].

MCFAs are obtained from edible fats such as coconut oil and milk fat by lipid fraction separation [21]. They are also present in palm oil (8%) and cuphea oil [22,23,24,25]. This group of fatty acids is composed of caproic (C6), caprylic (C8), capric (C10), and lauric (C12) acids. Coconut oil, palm oil, and their combination belong to the group of MCFAs [26]. SCFAs are saturated aliphatic organic acids that consist of one to six carbon atoms. The most abundant are acetate (C2), propionate (C3), and butyrate (C4), and these acids are produced in the gastrointestinal tracts of humans and animals [27,28]. They play different important roles in the development of intestinal epithelial cells. They modulate some processes in the gastrointestinal tract, such as electrolyte and water absorption, or regulate several leukocyte functions, including cytokine production [29]. SCFAs are known for their antimicrobial characteristics due to their ability to cross bacterial membranes in their undissociated form. Inside the bacterial cell they dissociate, resulting in a higher anion concentration and bactericidal effect [30].

In this review, current findings and prospects for further research on the poultry microbiome and feeds supplementation with SCFAs and MCFAs are summarized.

## 2. Natural Sources of SCFAa and MCFAa

The fatty acids most commonly used in diet supplementation for poultry to control microorganisms are formic acid, benzoic acid, citric acid, carboxylic acids (all SCFAs), and their salts, as well as some MCFAs, including caproic (C6:0), caprylic (C8:0), capric (C10:0), and lauric acid (C12:0). Coconut oil is a highly saturated oil (about 90%), and 60% of its total fatty acid composition is made up of MCFAs with a chain length of 6 to 12 carbon atoms [22] that are absorbed directly into the portal circulation without re-esterification in intestinal cells [31]. The MCFAs are partly independent of the carnitine transport mechanism into the mitochondria of the liver and are rapidly and exclusively oxidized for the production of energy [32]. Medium-chain fatty acids (MCFAs) are naturally found in many plant oils (palm, red palm, palm kernel, babassu, coconut, and murumuru) or dairy products [33,34]. Palm and red palm oils contain palmitic (42.9% and 41.96%, respectively) and oleic (40.28% and 40.6%, respectively) fatty acids in the largest proportion. Lauric and oleic acids are the top components of coconut (41.31% and 11.72%, respectively) and babassu (43.98% and 13.62%, respectively) oils [33]. However, the oil composition of the same lipid sources might show differences, as the extraction method might alter the amount of different fatty acids [35]. MCFAs and myristic acid (C14) can influence the immune function, so they play an important role in stimulating cell-mediated immunity. The hydrophilic and hydrophobic characteristics of MCFAs play a key role in their antimicrobial activity, i.e., they can cause lysis of the cell [35,36]. MCFAs also play an indirect role in the availability of nutrients for birds [37].

SCFAs are formed from polysaccharide, oligosaccharide, protein, peptide, and glycoprotein precursors by anaerobic micro-organisms. Carbohydrates are the most important SCFA progenitors [38]. SCFAs are carboxylic acids and have an aliphatic tail of two to six carbons. They can be produced naturally through host metabolic pathways (fermented by symbiotic microbiota) and by SCFAs-producing probiotics [39,40,41]. The beneficial microbiota of broilers produces SCFAs as acetic, propionic, or butyric acids [41]. SCFAs are potential mediators of the microbiota–gut–brain axis; thus, they might play a role in neurological processes [40,42]. Furthermore, they have anti-inflammatory, antitumorigenic, and antimicrobial effects [39]. In broiler feed, the most frequently studied SCFAs are acetate, propionate, and butyrate, with two, three, and four carbon molecules, respectively, in their chemical structure [41]. These three fatty acids account for more than 95% of the total SCFA group [43].

New products have been developed through the formation of calcium and/or sodium salt with fatty acids or the esterification of these acids prior to adding it to the feed. Esterification has an important advantage as the esterified SCFA and MCFA escape gastric digestion, thus reaching the small intestine where they can exert their effect [44,45]. When these acids, in salt or esterified form, are fed to animals, positive effects on the growth performance, intestinal microbial growth, and health status of the animals are seen [46]. The potential effect of SCFAs and MCFAs without any protection would be limited because of prompt absorption and/or metabolism in the gastric area.

## 3. Production Parameters

Rapid growth and a high meat production efficiency are essential for successful broiler chicken production. Due to genetic development, the present days’ hybrids have an extremely high growth potential which must be understood in order to meet the increasing demand for chicken meat worldwide. Improving production parameters is an important objective for producers. Multiple studies have analyzed the effect of coconut and/or palm oils on the production parameters of broilers. These studies focus on growth performance, body weight, weight gain, feed efficiency, mortality, feed intake, feed conversion, relative liver, heart, and breast weight.

The slaughter weight of broilers is commonly 2–2.5 kg. Nowadays, meat-producing broilers reach this market-ready weight within 35–45 days [47]. The Cobb 500 hybrid attains a slaughter weight of 2.5 kg at 37–38 days of age with a feed conversion of 1.5 kg/kg [48]. The study of Wang et al. [24] indicated that feeding MCFAs in the form of coconut oil does not improve growth performance but reduces fat deposition and favorably affects lipid profiles without impairing performance in broilers.

Coconut diet (1.5% coconut oil) significantly increased the body weight gain of Cobb chickens in the 1–21-day period (590 g) compared to other oil diets (fish, canola, and mixed fish + canola + coconut); however, this benefit disappeared by 42 days (2038 g) [49]. A similar result was found by Khosravinia [50] with 2 g/kg MCFA in the same period of life in daily weight gain. It was found that 0.25% lauric acid and 0.25% capric acid alone or in combination significantly improved live body weight and cumulative weekly gain [12]. Zimborán et al. [51] observed no differences between control and supplemented groups (5% coconut oil, 5% palm oil, 2.5% coconut + 2.5% palm oil) in terms of body weight from day 1 to day 28 of age. On day 42, live body weight was 2007.3 g in the coconut group, while it was 2013.1 g in the control. Several authors have reported that palm oil supplementation does not affect body weight and weight gain [52,53,54]. However, the study of Rahman et al. [55] revealed the negative effect of adding 4% palm oil to the diet on the growth parameters from the second and fourth week of the experiment. Zimborán et al. [51] described 2005.9 g body weight on day 42 with 5% palm oil in the feed; however, the group fed with mixed oil supplementation (2.5% coconut and 2.5% palm oil supplementation) reached a 1952.3 g body weight. This weight was not significantly less than the weight achieved by adding a single oil supplement to the feed. In agreement with these data, Khatun et al. [56] reported that Cobb-500 hybrids achieved a body weight of 2126.8 g by day 42 with 6% palm oil supplementation. Similarly, a 5% palm oil supplementation generated the same slaughter weight of the Hubbard Classic broiler control group on day 42 [55]. Khatun et al. [56] measured a gain of 1454.8 g with 6% palm oil supplementation between days 21 and 42, while 5% palm oil in the feed resulted in a 1430.4 g body weight gain [51]. In the similar period, from day 21 to day 42, Attia et al. [49] obtained 2038 g gain with 1.5% coconut oil added to the diet. This result exceeded the weight gain of 1446.3 g measured with 5% coconut oil supplementation [51]. The 2.5–2.5% mixed coconut and palm oil group showed the lowest body weight gain (1369.5 g) during the same growing period compared to the addition of pure coconut or palm oil [51]. Comparing the impact of short- and medium-chain fatty acids, no significant differences were found in the body weight and weight gain parameters [28] using pure MCFA and SCFA supplements alone or in combination.

Some studies have reported beneficial effects of oil supplements on the feed efficiency of broiler chickens [57,58], but controversial results have been reported too [59]. Khosravinia [50] presented the reduced mortality of broilers by a high dose (2 g/kg) of MCFAs. Furthermore, MCFAs were also beneficial in cases of high flock density (16, 18 birds/m^2^) by reducing the occurrence of foot morbidities.

Wang et al. [24] and Attia et al. [49] measured the feed intake and feed conversion between 21 and 42 days of life in Arbor Acres and Cobb hybrids. The coconut oil group (1.5% supplementation) in the study of Wang at al. had a lower total daily feed intake [24] than that found by Attia et al. [49] with the same supplementation; however, these values were more or less the same as the control values in the two experiments (Table 1). Similarly, Khosravinia [50] did not find significant differences between the control and experimental groups (1.5 and 2 g/kg MCFAs) in terms of feed intake. Rebolé et al. [60] found the same non-significant effect with palm oil (90 g/kg diet) supplementation. Birds with 5% coconut oil supplementation consumed a total of 2510 g of feed, 119.5 g per day, between day 21 and 42 [51]. The feed intake of Hubbard Classic [55] and Cobb 500 hybrids [56] supplemented with 5% and 6% palm oil, respectively, did not differ from the control. Birds supplemented with 5% palm oil, the mixed group (2.5–2.5% coconut and palm oil), and the control group consumed similar amounts of feed [51]. Feed conversion was not affected by MFCA supplementation, while body weight gain was 49 g higher in broilers fed with supplemented feed [59].

Considering feed conversion results, Wang et al. [24] and Attia et al. [49] showed more pronounced differences among groups supplemented with variable oil sources compared to the feed intake data. Wang et al. [24] observed a higher feed efficiency than Attia et al. [49] with the same amount of coconut oil supplementation (Table 1). The results of Zimborán et al. [51] are consistent with those of Wang et al. [24], with coconut and palm oil supplementation. Khatun et al. [56] observed that palm oil supplementation decreased feed conversion lower than 2.05 g/g [51]. A 1.5 g/kg MCFAs supplementation generated a higher feed conversion ratio than 2 g/kg MCFAs [50], while the cumulative weekly feed consumption and the cumulative feed conversion ratio were improved in broilers fed a high-MCFA diet [12].

The relative liver weight was 2.31% in the 1.5% supplemented coconut oil group of Attia et al. [49] compared to the results (1.5%) found by Zimborán et al. [51]. In the case of relative heart weight, the trend was similar. Attia et al. [49] described 0.55% relative heart weight in the coconut oil group, while Zimborán et al. [51] observed lower values (0.38%) in the groups supplemented with coconut and coconut + palm oils, respectively. The relative liver weight was not affected by the palm oil (90 g/kg diet) treatment in the work of Rebolé et al. [60]. Khatibjoo et al. [28] also failed to find differences in spleen, bursa, and thymus weight due to supplementation with MCFAs, SCFAs, and their combination. MCFA or SCFA supplementation affected neither the breast meat, the thigh meat yield and carcass weight, nor the protein and lipid percentage of breast and thigh [28]. The European Performance Efficiency Factor (EPEF) was found to be improved when 0.25% of lauric acid supplementation (324.6) or its combination with capric acid was utilized, respectively (323.8) [12]. EPEF was also improved by alpha-monolaurin (0.25 g/kg) [61] and 2% MCFA supplementation [50] in broilers, respectively.

The parallel use of MCFA and coccidiostats has positively influenced production results in broilers (weight at the end of the production cycle, feed intake, growth, and conversion rates) [62]. Fat, protein, and crude fiber digestibility was improved by adding MCFA with selected organic acids (propionic, fumaric) [63]. Zeits et al. [64] used a different ratio of C12 and C14 in broiler nutrition and did not find any differences in microbiota, intestine morphology, fat, or cholesterol content in meat or liver, but adding MCFA had a positive effect on the feed conversion and pectoral weight of broilers.

## 4. Meat Quality Parameters

Maintaining a high meat quality is essential for quality food and its longer shelf life. For meat quality testing, it is necessary to measure parameters such as pH, drip loss, color, kitchen losses (melting, cooling, and cooking), and tenderness [65,66,67,68].

In a recent experiment, 5% coconut oil and mixed oil supplementation (2.5–2.5% coconut + palm oil) resulted in the same pH 24 h after slaughter at 42 days of age, while it was noted to be lower when adding 5% palm oil in the diet [51] (Table 2). These results are in line with those of Souza et al. [69] and Khatun et al. [56] and correspond to the normal pH of broiler meat [70]. According to Jiang et al., [71] pH 24 varied between 6.01 and 6.06, which is consistent with the average final pH of broiler breast meat (6.03) [72]. A total of 2% coconut oil in the feed resulted in breast pH [73], which is not similar to the results of Zhang et al. [74] and Gao et al. [75] with sodium butyrate. Ogunwole et al. [76] measured the effect of 2% coconut oil and 2% palm oil in feed separately. The coconut group had a higher pH value than the palm oil group at 24 h. According to these data, palm oil might result in a lower pH than coconut oil, which, hypothetically, might be related to the different fatty acid composition of the two oils and the consequent difference in the fatty acid composition of the meat itself.

According to the literature data, different types of fat sources often cause changes in meat color. Abdullah et al. [77] found that the L* value for broilers slaughtered on day 42 was 53.35. Prayitno et al. [73] did not experience differences in color in groups with different percentages (0–2%) of coconut oil. The other color parameters were not affected by the supplementation. In the study of Ogunwole et al., [76] the L*, the a*, and the b* were measured to detect the color of the meat when 2% coconut oil supplementation was present in the diet. L* and b* values were higher; the other parameter(a*) was lower in the group fed the same amount of palm oil supplementation. A higher (6%) amount of palm oil in the feed resulted in color changes, as presented in Table 3 [56]. A 5% coconut oil or 5% palm oil supplementation resulted in similar color results. The same trend was observed in the mixed group (2.5–2.5% coconut and palm oil supplement) [51]. Sn1-monoglycerides, as a source of saturated short- and medium-chain fatty acids (SMCFA), yielded significantly increased L* and b* values in comparison with the control group. Based on the ∆E results (∆E > 5), referring to the visibility of color differences [78], Sn1 monoglycerides strongly modified the meat coloration [66].

The literature data on the water holding capacity/drip loss of meat are highly variable. In the study of Prayitno et al. [73], with different amounts of coconut oil supplementation, the water holding capacity of broiler meat was diverse (Table 4). A similar finding was obtained when the water holding capacity of broiler chickens fed with 2% coconut oil was higher than that of 2% palm oil [76]. Zimborán et al. [51] observed similar drip loss data in the 5% coconut and 5% palm oil groups. A total of 2.5–2.5% coconut and palm oil in the feed resulted in higher drip loss values. However, Khatun et al. [56] found that drip loss was only 3.06% in the group of 6% palm oil supplementation. The variability in these results is partially due to the differences in the analytical methods. However, irrespective of the different methods, it seems that different types of oils do not affect the water holding capacity of meat [79].

Kitchen losses refer to losses occurring during processing the meat in the kitchen. In our work, it refers to melting, frying/cooking, and cooling losses. Measuring kitchen losses is an important part of meat quality assessment and it comprises the loss of the mass of the meat due to freezing and thawing, as well as the losses during cooking and consequent cooling. Similar to the drip loss, this is a very important quality measure not only for customers but also for the processing companies. Most commonly, cooking loss is monitored in the existing research, and this parameter shows high variability according to the literature data. Thus, Soeparno [80] found that the cooking losses of broiler chickens at 6 and 7 weeks of age were 24.89% and 34.57%, respectively, and showed high individual variance. Somewhat lower values were reported by Prayitno et al. [73] regarding cooking loss without added oil in the diet, while a higher value was measured when 2% coconut oil supplementation was used (Table 4). It was also revealed that 2% palm oil inclusion in the diet caused a higher cooking loss than the same rate (2%) of coconut oil [76]. However, when palm oil was added in a higher inclusion rate (6%), lower cooking loss occurred compared to the 2% inclusion rate value [56]. In another experiment, overall kitchen losses were presented in groups with various different oil supplementation schedules. The 5% coconut oil inclusion in the diet resulted in higher kitchen loss than 5% palm oil in the feed, while mixed 2.5–2.5% coconut and palm oil supplementation produced the lowest value [51]. Based on these results, coconut oil supplementation has a negative effect on kitchen losses compared to palm oil.

Tenderness refers to how tough the meat is. It can be characterized by shear force and, in chickens, the normal value varies around 1.5–2.0 kg/cm^2^. Whether the diet was based on corn, milo, or wheat, the tenderness of the breast meat was in the range between 1.82 kg/cm^2^ and 2.19 kg/cm^2^ [81]. However, a number of scientific projects focused on oil supplementation reported much higher shear force data. For example, the values measured by Prayitno et al. [73] were the lowest for 2% coconut oil supplementation and the highest for 0.5% coconut oil (Table 5). Khatun et al. [56] recorded lower shear force in Cobb 500 broiler chicken breast meat when 6% palm oil was added to the diet. However, 2% palm oil or 2% coconut oil supplementation resulted in an increased value of this parameter, which means the meat became firmer due to additional oil [76]. The results of Zimborán et al. [51] are closest to those of Lyon et al. [81]. In this research, the shear force value of the group fed with 5% coconut oil or palm oil was lower than 2 kg/cm^2^, while it was higher in the samples of mixed 2.5–2.5% coconut and palm oil treatment.

The sensory attributes of chicken meat in the experimental medium-chain fatty acid group had significantly higher scores in terms of the total score, overall perception, and taste compared to the control group. In the organoleptic investigation, the cooked meat was juicier than the control. However, there were no differences in the case of ash, total protein, and moisture contents [82]. Dauksiere et al. [83] presented similar a trend: in the case of ash, protein content, and intramuscular fat, there were no significant differences between control and MCFAs groups. MCFAs, SCFAs, and the combination of these did not affect the protein content of the chicken meat, but the lipid percentage significantly differed from the control group [28]. Palm oil supplementation did not influence the texture (hard, juicy, fatty), odor, or flavor of the chicken meat [84]. The same trend was obtained in the parameters of juiciness, general acceptability, texture, and taste with 5% palm oil supplementation [85]. Different coconut oil supplementation did not affect the texture of the chicken meat. There were no significant differences in the case of taste and juiciness [73]. Similarly, no significant impact was observed in terms of juiciness, flavor, and texture based on 5 or 8% coconut supplementation [86]. There was no difference in the impact of palm kernel or coconut oil supplementation on parameters like texture, overall acceptability, juiciness, and flavor [76].

Based on the literature, short-chain fatty acids have a positive effect on meat quality parameters, which ensure the enjoyment value of meat and provide a basis for quality production. The performance of organoleptic tests may provide greater insight into consumer feedback in the future.

## 5. Molecular and Biochemical Parameters

Short- and long-chain fatty acids might cause changes in several biochemical processes and parameters in poultry. They are thought to be involved in energy metabolism signaling. Lipid parameters are highly variable in poultry; therefore, the absolute values available in the literature from different research groups cannot be easily compared. However, Khatibjoo et al. [28] showed that MCFAs, SCFAs, and the combination of these can decrease the serum cholesterol concentration of broilers. Similar results were found by Khatun et al. [56] when the effects of 6% pure palm oil supplementation were compared with mixed oil supplementation. The mechanism in the background is not totally clear, but SCFAs, for instance, are able to reduce the activity of certain enzymes, such as 3-hydroxy-3-methylgltaryl-CoA synthase and 3-hydorxy-3-methylglutaryl-CoA reductase. As these are key enzymes for cholesterol synthesis in the liver, this effect is likely to lower the blood cholesterol concentration.

Khatun et al. [56] also used 6% palm oil supplementation; they detected 0.68 mmol/L of blood plasma TG (triglycerides) and 3.26 mmol/L cholesterol. Less palm oil (4% palm oil + 2% sunflower oil, 2% palm oil + 4% sunflower oil) resulted in a decrease in TG (0.51, 0.55 mmol/L) and cholesterol (2.67, 2.64 mmol/L). However, in the study by Attia et al. [49], 1.5% coconut oil supplementation did not affect the plasma lipid profile of 42-day-old broiler chickens. Same results were published by Wang et al. [24] with 1.5% coconut oil supplementation. In the experiment of Zimborán et al. [51], significant changes were found in the plasma TG and cholesterol concentration due to 5% coconut, palm, or mixed (coconut + palm) oil inclusion in the diet, with the exception of the TG concentration in the coconut-oil-treated group. This latter value was significantly higher than the control, which is contradictory to the other research findings.

As fatty acids are good electron donors in the mitochondrial electron transfer, reactive oxygen species might be released due to their oxidation. The excessive accumulation of MCFAs might result in increased ROS production and consequently lead to lipid peroxidation. In certain conditions, these fatty acids might induce apoptosis and parallel the modification of the glutathione level and generate reactive oxygen species. Certain MCFAs have a protective activity against oxidative stress. When 2, 4, and 6% palm oil were added to the feed in the work of Long et al. [87], the blood plasma malondialdehyde (MDA) concentration, an oxidative stress indicator, was 7.93 nmol/mL, 6.44 nmol/mL, and 6.44 nmol/mL, respectively, on day 42, while the activity of an antioxidant enzyme, the glutathione peroxidase (GSHPx) in the blood plasma, was 765.3 U/mL, 789.3 U/mL, and 880.5 U/mL. In another experiment with 5% coconut oil, 5% palm oil, or their combination, some reduction was also found in the MDA concentration, while the differences in the GSHPx activities were not consistent. Similar results were reported by Attia et al. [49] with 1.5% coconut oil supplementation, where the MDA concentration was markedly reduced. So, in general, the higher the SCFA and MCFA in the diet, the better the oxidative status in the different tissues.

Sodium butyrate could lower the MDA level while increase the serum superoxide dismutase enzyme activity in chickens in normal conditions [88]. A total of 1 g sodium butyrate/kg (which is at the highest end of regular inclusion rate range) in the diet did not influence the MDA amount [75] and did not cause any change in the oxidative status of chicken meat [78]. However, in the work of Saleh et al. [61], with a potential MCFA additive, alpha-monolaurin (0.25 and 1 g/kg diet supplementation, respectively), the MDA levels were significantly reduced in birds.

Altogether, there is no clear conclusion for antioxidant defense parameters regarding the supplementation of short- and long-chain fatty acids. However, as the use of fatty acids does not have any proven negative effect on lipid peroxidation processes, their administration is preferable due to their other beneficial effects.

## 6. Microbial Attributes of SCFAs and MCFAs

Enteric diseases are major concerns in the poultry industry due to production losses, increased mortality, reduced bird welfare, and increased risk of the contamination of poultry food products. A reduction in pathogenic microorganisms in the digestive tract decreases the contamination of poultry products. Salmonella and Campylobacter are considered major public health burdens worldwide, and epidemiological studies demonstrate that poultry are known to be one of the main reservoirs for these zoonotic pathogens; therefore, poultry products are frequent sources of these pathogenic agents.

According to the literature data, MCFA inhibit bacterial toxin production and the expression of other virulence factors by interfering with signal transduction [89,90]. The effect of these acids is both bactericidal (killing) and bacteriostatic (growth-inhibiting) depending on the concentration, synergism among them, and target bacterial strain [44,91].

The details of the mechanism of the antimicrobial effects of SCFA and MCFA or the synergistic activities between these acids are not perfectly revealed [1,92,93]. It is assumed that due to their lipophilic character, in undissociated form, they can penetrate the microbial cell wall and deteriorate the cell membrane, causing increased permeability and cell leakage, causing the death of bacteria [17]. It is also assumed that within the cell these acids, pH might be reduced, which can alter the activity of the cytosolic enzymes [1].

Mathis et al. [94] proved that the combination of organic acids and MCFA significantly reduced clinical symptoms of diseases in artificial necrotic enteritis of broilers. They showed in broilers infected with viral malabsorption syndrome (MAS) that adding SCFA and MCFA together in feed increased broiler growth and resulted in higher broiler weight at the end of the production cycle. How this directly impacts viruses is not known, but it is considered that SCFA and MCFA together have a synergistic effect on bacteria, whereby MCFA damages microorganisms’ cell walls, thus allowing SCFA access into the bacterial cytoplasm to produce an antibacterial effect. Butyrate appears to be bactericidal and can stimulate villi growth.

### 6.1. Salmonellosis

*Salmonella typhimurium* and *enteritidis* are often detected in poultry and foods, being associated with several human foodborne outbreaks [95]. Formic acid has strong antimicrobial effects in poultry, as reviewed by Ricke et al. [27]. Feeding formic acid (4 kg/ton) for 6 weeks resulted in no recovery of *Salmonella* from ceca in broilers compared to the control prevalence of 17% [27]. Deschepper et al. [96] showed that MCFA at 0.8 and 1.2 g/kg feed reduced the invasion of Salmonella enteritidis in the intestine of broilers. Van Immerseel et al. [97] found that MCFA reduces the colonization and invasion of Salmonella enterica shortly after infection in chickens, and a similar result together with the increased transcription of antimicrobial peptide was revealed. Evans et al. [98] reported that the addition of MCFA in turkey diet could lower the colonization of Salmonella in the early period of life.

### 6.2. Campylobacteriosis

Broiler chicken is a natural host for *Campylobacter* spp., and contaminated poultry meat products are major sources for transmitting pathogenic *Campylobacter* strains to humans [99,100]. MCFA shows marked anti-*Campylobacter* activity in vitro. In vivo studies indicated that the supplementation of MFCAs may be a promising tool either for the the prevention of or reduction in *Campylobacter jejuni* colonization in commercial broiler flocks [60]. Solis de los Santos et al. [101] evaluated the effect of caprylic acid on the cecal *Campylobacter jejuni* colonization at experimental infection in broilers and found that the dose of 0.35% in a regular starter diet was more efficient compared with the spectrum of other five higher supplementations up to 1.4%.

Molatová et al. [102] examined the effects of the supplementation of feed with a coated or non-coated mixture of fatty acids (*caprylic* and *capric* acid) in broiler chickens experimentally infected with *Campylobacter jejuni*. Their results indicated that MCFA, and especially their coated form, may help to reduce the level of colonization of chicken intestine by *C. jejuni*. In the study of van Gerwe et al. [59], supplementing the feed with a mixture of MCFAs (C8–C12) decreased the susceptibility of broilers for *Campylobacter* colonization.

Drinking water is a prominent source of horizontal *Campylobacter* transfer [103]. Results on the application of MCFA in drinking water are contradictory. Metcalf et al. [104] reported that treatment with caprylic acid in water had an inconsistent effect on the intestinal *Campylobacter* counts, whereas the drinking water application of MCFA was effective in combating *Campylobacter* colonization in poultry [105]. Although *Campylobacter* colonization and transmission was not reduced by adding an emulsion of a mixture of caproic, caprylic, capric, and lauric acids to the drinking water of broiler chicks, they were found to be less susceptible to the colonization of *Campylobacter jejuni*, which seems to be the result of the reduced survival of the bacteria in drinking water. Considering these results, the benefit of water applications of MCFA is thought to be the reduced probability of *Campylobacter* invasion in the digestive tract of a bird and then the transmission of the pathogen throughout a flock.

### 6.3. Clostridiosis

*Clostridium perfringens* is among the five most frequent pathogens causing foodborne diseases in humans. This microbe is a normal member of chickens’ intestinal microbiome as it is acquired from the environment, like in water, food, litter, etc. [97]. Certain strains might produce toxins, adhesins, special proteolytic, and collagenolytic enzymes which can alter the healthy structure of the intestinal mucosa, altering the physiological status of the gastrointestinal tract and resulting in necrotic enteritis. Supplementation with MCFA-containing fats and oils in feed, using coconut and palm oil, was found to be effective against *Clostridium perfringens*, mainly as a preventative treatment [25]. However, coconut oil and palm oil did not affect the production parameters of broilers during *Clostridium perfringens* challenge [37]. Hovorková et al. [33] found in vitro inhibitory effect of palm kernel oil against clostridia. Similarly, the data of Shilling et al. [106] indicated that lauric acid (C12) can significantly inhibit the growth of *Clostridium difficile*, while *Capric* (C10) and *caprylic* acid (C8) were less effective. However, in an in vivo experiment, Clostridium perfringens was not inhibited by lauric acid in the work of Yang et al. [107], while Abdelli et al. [108] have found the improved histomorphology of the intestinal mucosa and reduced clostridia excretion in the excreta. Sodium lauryl lactylate was also found to prevent and inhibit the colonization of *Clostridium perfringens*. Qi et al. [36] showed that microencapsulated C8–C12–C14, C12–C14, or C10–C12 combinations displayed efficient antibacterial effects on the wild isolated avilamycin-resistant strains (CP-MZ1, *C. perfringens* type G strains, P-C8-1, *C. perfringens* type A strains), suggesting that the microencapsulated mix of different MCFAs together with myristic acid has potential for the clinical treatment of necrotic enteritis.

### 6.4. E. coli

*E. coli* is a normal inhabitant of chicken gut microbiome. Though many of the *E. coli* are not pathogenic, some have acquired virulence factors and can cause colibacillosis, which manifests in inflammatory process in different organs of the birds. As such, this is the most common and economically detrimental bacterial disease in avian species worldwide.

Short- and medium-chain fatty acids are thought to be beneficial to reduce the effects of coli pathogenicity due to their antimicrobial effects. However, the results are quite contradictory. No difference was found in the number of coliforms (TCC) in the digesta of broilers treated with coconut and/or palm oil in feed [25]. Similar results were reported by Jadhav et al. [12] with lauric and/or capric acid supplementation, as the TCC was slightly, but not significantly, reduced. However, Skřivan et al. [109] revealed that the supplementation of caprylic acid reduced coliform count in broilers. TCC was also significantly lower due to an MCFA mix supplementation compared to the control within the initial 28 days of the fattening period [110]. According to Ripon et al. [111], the antimicrobial activity of fatty acids needs time to develop because in their broiler experiment, TCC count was not affected by a mixture of medium-chain fatty acids in the first 21 days of growth, while by day 42, it was reduced significantly compared to the control values.

### 6.5. Coccidiosis

Avian coccidiosis is an infectious enteric disease caused by protozoan parasites of the genus Eimeria with a major impact on poultry production. Although coccidiosis may be subclinical, mucosal intestinal lesions can compromise the performance of broilers [41]. Coccidia are host-specific in poultry and the different species are likely to infect different locations of the intestine, i.e., *E. acervulina* and *E. maxima* are the most prevalent species in chicken, causing infection in the small intestine, while *E. tenella* is localized in the ceca. There are several studies which have outlined the positive effect of SCFAs and MCFAs in coccidiosis challenge. According to these results, modifying microbiome composition’s anti-inflammatory effects and improving the gut barrier are the probable mechanisms behind the veneer. Thus, a higher number of goblet cells, helping to create an intestinal barrier in the mucosa, and a reduced villus/crypt ratio, as well as increased lactic acid bacteria count, were found by Sadurni et al. [112] in the intestine of infected birds when a mixture of fatty acid salts distilled from coconut oil was added to their feed. It was proven by Place et al. [113] that butyrate inhibits nuclear factor kappa B activation, and, consequently, this results in the reduced expression of proinflammatory cytokines. This effect of tributyrin (a glycerol ester of the butyrate) was confirmed by Hansen et al. [114] as the gene expression of certain enzymes and proteins (junctional adhesion molecule, liver-enriched antimicrobial protein 2, mucin 2) related to gut immunity has changed due to butyrate supplementation, especially in birds with coccidiosis challenge. It is also important that though there are several types of butyrate feed additives, the most efficient forms are the glycerol esters, like tributyrin, because they cannot be absorbed before reaching the small intestine.

## 7. Implications for Future Research

According to the results of several decades’ research with short- and medium-chain fatty acids in broiler chicken diet, it is clear by now that these acids may improve the performance of chickens directly as well as indirectly. However, the results are not consistent when we consider the form of supplementation. When single fatty acids, like lauric or myristic acid, are used, it is obviously easier to evaluate their effects [61]. However, a blend of MCFAs and SCFAs are commonly used in the form of natural vegetable fat sources, like coconut oil or palm oil composed of numerous different fatty acids. Thus, in general, the more complex the composition of a fat source is, the more complicated it is to evaluate its effects. It is also reasonable to mention that when a blend of fatty acids is used, they might interact (synergism or antagonism), which makes the identification of the mechanism behind the effect even more difficult [93]. From a practical point of view, it is more reasonable to use natural vegetable oils in broiler nutrition. In this case, not only is the possible biological activity of the individual fatty acids included but their energy value should also be considered, which makes the evaluation of the literature data even more difficult.

As we have highlighted several times in the present article, research results even with the same fatty acid supplementation may vary. Therefore, the need for further research with pure fatty acid supplements and natural vegetable oils in this field is inevitable. The antimicrobial or bacteriostatic effects of MCFAs and SCFAs are likely the most prosperous side of the story. There are still so many questions that we cannot answer in this field. Since microbiome is a key factor in nutrient utilization and immune status of the birds, it is essential to clarify how the different members of the microflora can be supported or inhibited by these fatty acids and their blends and thus how these supplements can help to stabilize the microbiome composition and maintain eubiosis.

## 8. Conclusions

Nutritional strategies to improve gut health are under extensive research to reduce antibiotic use in poultry production. The previous and current literature proved that as alternatives for antibiotics, the use of SCFA and MCFA has important health and performance benefits in broilers both at experimental and commercial levels. When these acids, in salt or esterified form, are fed to animals, positive effects on the growth performance, intestinal microbial growth, and health status of the animals are seen. Although the full mechanism of action of SCFA and MCFA is not well-known, broad-spectrum activity has been demonstrated against Gram-positive and Gram-negative pathogenic bacteria such as *Salmonella*, *E. coli*, and *Clostridium* spp., making them a viable solution to reduce the use of antibiotics. They also have synergistic effects when used together and can thus reduce the magnitude and duration of treatments. However, when using natural oil sources rich in SCFAs and/or MSCFAs, the results are often contradictory as the fatty acid composition of these feed additives might be variable. In these blends, only the proportion of the different fatty acids are measured, while we do not really know what their absolute concentration is in the oil and therefore in the diet. Thus, results from the experiments working with single or blended fatty acids are not easily comparable. Also, the potential effect of SCFA and MCFA without any protection would be limited because of prompt absorption and metabolism—or both—in the stomach or in the small intestine, while their bacteriostatic activity can be realized in the ceca and in the lower gut.

## Figures and Tables

**Table 1:** Feed intake and feed conversion changes by fatty acid supplementation.

**Supplementation**	**Feed Intake (g) ^1^**	**Work of**
1.5% coconut oil	2914.8 ^T^	[24]
1.5% coconut oil	3064 ^T^	[49]
1.5 and 2 g/kg MCFA	82.06; 83.09 ^D^	[50]
5% coconut oil	2510 ^T^	[51]
2.5 + 2.5% coconut and palm oil	2760 ^T^	[51]
5% palm oil	2860 ^T^	[51]
5% palm oil	3064 ^T^	[55]
6% palm oil	2530.7 ^T^	[56]
**Supplementation**	**Feed Conversion (g/g)**	**Work of**
1.5% coconut oil	1.82	[24]
1.5% coconut oil	1.52	[49]
2.5 + 2.5% coconut and palm oil	1.79	[51]
6% palm oil	2.05	[56]
1.5 and 2 g/kg MCFA	1.9; 1.79	[50]

^1^ T: total feed intake, D: daily feed intake.

**Table 2:** Changes in pH after 24 h by different fatty acid supplementation.

Supplementation	pH 24 h	Work of
5% coconut oil	5.98	[51]
2.5 + 2.5% coconut and palm oil	5.98	[51]
5% palm oil	5.89	[51]
6% palm oil	5.80	[56]
4 + 2% palm and sunflower oil	5.79	[56]
2 + 4% palm and sunflower oil	5.78	[56]
2% coconut oil	6.25	[73]
0.4 g/kg sodium butyrate	5.88	[74]
1 g/kg sodium butyrate	5.50	[75]
2% coconut oil	5.91	[76]
2% palm oil	5.35	[76]

**Table 3:** Changes in color parameters by different fatty acid supplementation.

Supplementation	L*	a*	b*	Work of
5% coconut oil	63.04	11.18	9.38	[51]
2.5 + 2.5% coconut and palm oil	62.87	10.98	9.39	[51]
5% palm oil	63.58	11.58	8.80	[51]
6% palm oil	50.31	3.93	14.21	[56]
4 + 2% palm and sunflower oil	49.36	3.94	14.22	[56]
2 + 4% palm and sunflower oil	48.20	4.71	14.24	[56]
3.0 g/kg Sn1-monoglycerides	63.80	5.60	8.99	[78]
2% coconut oil	45.35	8.31	5.45	[76]
2% palm oil	52.31	6.02	5.45	[76]

**Table 4:** Drip and cooking loss values from different fatty acid supplementation experiments.

**Supplementation**	**Tenderness**	**Work of**
5% coconut oil	10.33	[51]
2.5 + 2.5% coconut and palm oil	11.15	[51]
5% palm oil	10.44	[51]
6% palm oil	3.06	[56]
4 + 2% palm and sunflower oil	3.08	[56]
2 + 4% palm and sunflower oil	3.09	[56]
0.5% coconut oil	35.84	[73]
1% coconut oil	35.85	[73]
1.5% coconut oil	40.99	[73]
2% coconut oil	42.21	[73]
2% coconut oil	10.21	[76]
2% palm oil	39.87	[76]
**Supplementation**	**Cooking Loss %**	**Work of**
0.5% coconut oil	25.32	[73]
1% coconut oil	25.54	[73]
1.5% coconut oil	19.14	[73]
2% coconut oil	18.87	[73]
2% coconut oil	17.98	[76]
2% palm oil	29.32	[76]
6% palm oil	24.83	[56]
4 + 2% palm and sunflower oil	24.85	[56]
2 + 4% palm and sunflower oil	24.86	[56]
5% coconut oil	19.32	[51]
2.5 + 2.5% coconut and palm oil	15.47	[51]
5% palm oil	18.74	[51]

**Table 5:** Tenderness results from different fatty acid supplementation experiments.

Supplementation	Tenderness kg/cm^2^	Work of
5% coconut oil	1.93	[51]
2.5 + 2.5% coconut and palm oil	2.10	[51]
5% palm oil	1.80	[51]
6% palm oil	1.20	[56]
4 + 2% palm and sunflower oil	1.19	[56]
2 + 4% palm and sunflower oil	1.19	[56]
0.5% coconut oil	5.56	[73]
1% coconut oil	5.28	[73]
1.5% coconut oil	4.34	[73]
2% coconut oil	3.38	[73]
2% coconut oil	6.63	[76]
2% palm oil	5.67	[76]

## Data Availability

Data sharing not applicable.

## Sample Availability

Not available.
